# Guidelines for quantifying leaf chlorophyll content via non‐destructive spectrometry

**DOI:** 10.1002/aps3.11610

**Published:** 2024-08-03

**Authors:** Giancarlo M. Chiarenza, Eve Slavich, Angela T. Moles

**Affiliations:** ^1^ Evolution & Ecology Research Centre, School of Biological, Earth and Environmental Sciences, UNSW Sydney 2052 New South Wales Australia; ^2^ School of Molecular and Life Sciences Curtin University Bentley 6102 Western Australia Australia; ^3^ Mark Wainwright Analytical Centre, UNSW Sydney 2052 New South Wales Australia

**Keywords:** chlorophyll degradation, chlorophyll estimation, hyperspectral index, refrigeration, sampling effort

## Abstract

**Premise:**

Leaf chlorophyll is a fundamental bioindicator used in several fields; however, we lack clear guidelines for optimizing sampling efforts and producing comparable studies.

**Methods:**

We estimated the leaf chlorophyll content of 10 plant species using nondestructive spectrometry methods. We stored half of the leaves at 4°C and half at room temperature under similar light levels to assess the role of storage in the chlorophyll degradation rate.

**Results:**

The chilled mature leaves maintained a chlorophyll content within 5% of the original value for ~1.5 d, while the chlorophyll content of unrefrigerated mature leaves decreased rapidly, indicating that their chlorophyll content should be measured within 4 h. When refrigerated, the chlorophyll content of the expanding leaves remained within 5% of the original level for at least 5 d, but we suggest analyzing them within 3 d. In mature leaves, 73% of the variation in chlorophyll content is at the species level, 15% is between individuals, and the variation within leaves is negligible (<1%). Measuring one mature leaf from eight individuals was sufficient to provide a species chlorophyll estimate within 5% of the true value at least 80% of the time.

**Discussion:**

We advise researchers to prioritize sampling more individuals rather than repeating measures within leaves or individuals. Our findings will help researchers to optimize their time and research efforts, and to obtain more robust ecological data.

Leaf chlorophyll content is used as an indicator for many biological and ecological traits, such as photosynthetic rate (Curran et al., 1990; Palta, 1990; Croft et al., 2017), physiological status (e.g., health level, stress, stage of growth) (Curran et al., 1990; Gitelson et al., 2003; Perez‐Bueno et al., 2019), nitrogen content (Ali et al., 2017; Sonobe and Wang, 2017), and productivity and carbon dynamics (Whittaker and Marks, 1975; Zagolski et al., 1996; Li et al., 2018; Croft et al., 2015, 2020). Chlorophyll content can be assessed through spectroscopy, making the process faster than chemical extraction (Gitelson et al., 2003); however, we have no information about how much sampling is required to adequately capture spatial variation (i.e., within leaves, between leaves, or between individuals), or how soon after harvest the leaves must be analyzed for the chlorophyll estimation. We therefore do not know exactly where and when to target our sampling efforts. Our overarching goal in this study is to quantify the spatial and temporal variation in leaf chlorophyll content. We hope this work will make future studies more efficient, reliable, and comparable.

Chlorophyll gradually degrades after leaves are harvested (Anderson and Rowan, 1966; Xiao et al., 2022); therefore, our first goal was to determine how long after harvest the leaves should be measured to return chlorophyll values within 5% of the initial leaf mean. For ex situ analyses, researchers need to strike a balance between minimizing the number of trips to field sites to collect leaf samples and minimizing the amount of time elapsed between sample harvest and measurements (Hendry et al., 1987; Funamoto et al., 2002). Despite the potential impact of the post‐harvest period on chlorophyll measurements, the time elapsed between the harvest and the analysis of leaves is often unspecified in published studies (Gamon and Surfus, 1999; Haboudane et al., 2002; Sims and Gamon, 2002; León et al., 2007). Understanding chlorophyll degradation trends will help researchers make more accurate and robust measurements.

Second, we investigated the extent to which keeping the leaves cool can prolong the operational window for leaf chlorophyll quantification. Chlorophyll molecules decompose at a slower rate at lower temperatures (Steet and Tong, 1996), so refrigeration may allow samples to be measured several hours or even days after harvest.

Third, we asked to what extent leaf traits are related to the chlorophyll degradation rate (i.e., the amount of chlorophyll decayed per unit of time). We focused on leaf thickness, surface area, leaf mass per area (LMA), and leaf dry mass content (LDMC). Harvested leaves are more susceptible to environmental stresses, which increase the concentration of reactive oxidative agents that can degrade chlorophyll structure (Yamauchi, 2015); thus, increased exposure of post‐harvest leaves to environmental stresses can increase the physiological degradation rate of chlorophyll (Yamauchi, 2015). Thicker leaves tend to be more protected from external agents (Gonzalez et al., 2002; Leigh et al., 2012; Monteiro et al., 2016); therefore, we would expect thicker leaves to have a slower post‐harvest degradation rate than thinner leaves. Larger leaves have a larger surface area susceptible to stresses (Baldwin and Schmelz, 1994; Yates et al., 2010; Wright et al., 2017), which is particularly true when leaves have been detached. For this reason, the chlorophyll in larger leaves is expected to degrade faster than in smaller leaves. Leaves with a high LMA are usually thicker and/or denser (Niinemets, 1999; Wright and Cannon, 2001); therefore, similar to the effect of leaf thickness, we expect LMA to be inversely proportional to chlorophyll decay. The LDMC is positively correlated with the amount of structural and defense compounds, such as cellulose, lignin, and hemicellulose (Fortunel et al., 2009); therefore, we expect leaves with higher LDMC to have a slower chlorophyll decay rate. Our quantification of the relationship between these traits and the chlorophyll degradation rate will help researchers prioritize which types of leaves to process first.

Our fourth goal was to quantify the variation in chlorophyll content within leaves, between leaves of the same individual, between leaves of individuals of the same species, and between species to determine the number of leaves required to estimate the chlorophyll content to within 5% of the true mean. Li et al. (2018) and Lichtenthaler et al. (1996) reported greater variability in chlorophyll content between species than within; however, there is no current consensus regarding the number of leaves or individuals that should be sampled to characterize the chlorophyll content of a species. In some studies of leaf chlorophyll content, only one leaf per plant is sampled (Gamon and Surfus, 1999; Haboudane et al., 2002), while other studies have sampled three leaves per plant (Datt, 1999; León et al., 2007), or the quantity is variable (Gitelson and Merzlyak, 1997; Sims and Gamon, 2002; Gitelson et al., 2003). The number of individuals sampled per species also varies widely between studies (Datt, 1999; Haboudane et al., 2002; León et al., 2007). Although intra‐leaf variation has been studied (Borsuk and Brodersen, 2019), the number of observations per leaf used in spectroscopy is often unmentioned or limited to one (Gitelson and Merzlyak, 1997; Croft et al., 2015). This means previous researchers might not have chosen the correct minimum number of replicates or might not have allocated their efforts optimally. Insufficient sampling effort would not accurately estimate the mean value of a species, while collecting too many replicates would waste resources.

## METHODS

### Sample collection

Fieldwork for this study was done in two parts. The chlorophyll degradation rate study took place in June 2021 in Myall Lakes National Park (New South Wales, Australia; 32°24′04″S, 152°28′19″E; EPSG:4326 WGS84). The chlorophyll variation study took place between September 2021 and April 2022 in three locations in Sydney, Australia: Centennial Park (33°53′56″S, 151°14′02″E), the Mary O'Brien Reserve (33°54′22″S, 151°12′29″E), and the Dunningham Reserve (33°55′05″S, 151°15′40″E).

We selected a relatively homogeneous patch of vegetation in each study site and sampled the most abundant species by cover with at least 10 mature (30 in special cases, see below) and 10 expanding leaves per plant, where mature leaves were longer than 1 cm (the size required to facilitate accurate spectroscope measurements). When moving to a new patch, if the most abundant species was one already sampled, we chose the second most abundant to sample, and so on. At the end of the sampling, herbs were not represented, so we selected the most abundant suitable herbaceous species to include to ensure we incorporated different growth forms (the species list can be found in Appendix S1; see Supporting Information with this article). For the main fieldwork, 21 species were sampled, including three herbs, eight shrubs, and 10 trees (sensu Royal Botanic Gardens and Domain Trust, 2023).

Individuals were sampled along a transect whose direction was chosen via a random number generator. If the chosen direction required us to enter a different ecosystem, we chose a new direction. We collected the closest suitable individual we encountered along the transect. Every time an individual was sampled, a new direction was chosen until enough individuals were sampled. We reduced our chances of sampling clones by sampling individuals of the same species separated by at least one canopy or 1 m, whichever was the largest. When we could not find enough suitable individuals within a single site, we chose a different site with similar characteristics to continue the sampling procedure. We collected four individuals per species to test for chlorophyll decay, five individuals per species to measure leaf traits (thickness, surface, LMA, and LDMC), and 10 individuals per species to quantify chlorophyll variation.

Leaves were selected first by picking a random compass bearing and selecting the closest branch to this bearing from the middle of the plant. To select leaves to sample, we started from the growing tip of the branch, skipping the most external and moving down the branch away from the growing tip until we found a suitable leaf, i.e., with no major signs of herbivory, disease, discoloration, or other issues that might hamper spectrometric analyses. Suitable expanding leaves were half (±15%) the length of the average mature counterpart. Leaves were selected independently of sun exposure to allow for natural variation in leaf characteristics, and in a number that was both reasonable and relevant for the research question. Each time a leaf was sampled, a new direction was chosen. We selected four mature and four expanding leaves per individual for the chlorophyll decay study, and five mature and five expanding leaves for trait measurement, following guidelines from Pérez‐Harguindeguy et al. (2013). For the chlorophyll variation assessment, we selected 10 mature and 10 expanding leaves from seven individuals per species, and 30 mature and 10 expanding leaves from three individuals per species.

Immediately after being collected, all leaves were placed in resealable bags and stored inside a cooler bag with ice bricks until we reached the lab facility. The leaves were brought to the lab facility within 30 min of harvest for the chlorophyll degradation experiment and within 3 h for the chlorophyll variation study. Leaf thickness, leaf fresh mass, and leaf surface were assessed within 4 h of collection. For the chlorophyll degradation experiment, the mature and expanding leaves were randomly assigned to being (a) stored in a glass door refrigerator at 4°C, or (b) stored on a bench at room temperature (approximately 20–22°C). Storing leaves in a glass door refrigerator allowed for similar natural light exposure during the day/night cycle. In the chlorophyll variation study, leaves were kept at 4°C in a standard refrigerator until the analyses were performed. Leaves to be refrigerated were left in slightly open resealable bags to avoid them falling from the grids. Leaves stored at room temperature were instead removed from the bags.

### Leaf analyses

We distinguished between expanding and mature leaves in our analyses, because even within the same species, expanding and mature leaves can differ in morphology, chemistry, and chlorophyll content (Kursar and Coley, 2003; Barton et al., 2019). The impact of leaf age on chlorophyll content has rarely been studied, as most previous work only considers mature leaves (Yoder and Pettigrewcrosby, 1995; Haboudane et al., 2002; Li et al., 2018).

The spectral signature of each leaf was obtained using a portable spectrometer (Ocean Optics USB2000+; now Ocean Insight, Orlando, Florida, USA), with an Ocean Optics PX‐2 for a light source, as well as an Ocean Optics WS‐1‐SL Spectralon as the white standard and black felt as the dark standard. The software used to perform the spectrometry was Ocean Optics SpectraSuite version 2.0. We set a measurement angle of 45° using an anodized probe holder to isolate the sample from external light, a measurement distance of 1 cm, and an integration time of 100 ms; 10 scans were performed to generate the average for each sample (Dalrymple et al., 2015). The spectral signatures were analyzed using the R package pavo2 version 2.7 (Maia et al., 2019). The diffuse reflectance interval was considered between 400–800 nm, smoothed with a 0.25 span using the function *procspec*, and the negative values were adjusted by adding the minimum value to the overall signature. From the elaborated spectra, we calculated the modified simple ratio “mSR705” to estimate the total chlorophyll content index using the function *vegindex* from the R package hsdar version 1.0.3 (Sims and Gamon, 2002; Lehnert et al., 2019).

In the operational window study, each leaf was divided into six sections (bottom, middle, top; left, right), and the center of a random section was scanned. Throughout the experiment, the same point was maintained when rescanning the leaf. We avoided scanning prominent veins and damaged parts on the leaf surface. After the first scan, the leaves were scanned at the closest possible timepoint after 0.5 h, 1 h, 2 h, 4 h, 8 h, 16 h, 32 h, 64 h, and 128 h from the initial scan. We randomly rearranged the position of the leaves within each storage type daily.

In the chlorophyll variation experiment, the leaves were scanned once. We first selected a subsample of leaves to be scanned in all three sections (bottom, middle, top), randomly choosing the left or right side. We estimated the chlorophyll content only on the middle section (for ease of measurement) for the remaining samples, randomly choosing the side.

Leaf traits were assessed once, at the beginning of the experiment. Leaf thickness was measured with a Mercer dial gauge micrometer (d = 0.01), sampling near the middle section of a leaf on a randomly chosen side, and avoiding main veins and damaged spots. The leaf surface area excluding the petiole was measured using a flatbed scanner (Canon LiDE 220; Canon, Tokyo, Japan) at 300 dots per inch (dpi) and then analyzed with the R package LeafArea version 0.1.8 (Katabuchi, 2015). The leaf dry mass was measured using an analytical balance (Mettler Toledo XS105, d = 0.01 mg; Mettler Toledo, Columbus, Ohio, USA) after drying the leaves in an oven at 60°C for 72 h. The LMA was calculated as the ratio between the leaf dry mass and the leaf surface area (Pérez‐Harguindeguy et al., 2013), while the leaf dry matter content was the ratio of the leaf dry mass and fresh mass (Pérez‐Harguindeguy et al., 2013).

### Statistical analyses

All statistical analyses were run in R version 4.3 with RStudio version 2023.03 (Posit Team, 2023; R Core Team, 2023). Reflectance‐based chlorophyll content estimates and leaf traits were log‐transformed (base 10), and time (in h) transformed using log_10_ + 1, to satisfy statistical assumptions and highlight relative changes.

#### Chlorophyll decay study and suitable operational window

Leaf chlorophyll decay was calculated by running a linear mixed model using lme4 version 1.1‐27.1 without analytical derivatives (Bates et al., 2015). The response variable was chlorophyll content, while the fixed effects were the three‐way interaction between the elapsed time since the first scan, the leaf growth stage (expanding vs. mature), and the storage type (room temperature vs. refrigeration). Random effects included species and individuals, and we included random slopes for time and storage between species (i.e., chlorophyll ~ time × leaf growth stage × storage + (1 + storage + time | species) + (1 | individual)). We added random effects for species as they might respond differently to chlorophyll decay kinetics due to species‐specific adaptations, e.g., water availability or heat stress. We used emmeans version 1.6.3 to extract the estimates of slopes and confidence intervals from 1000 iterations of the parametric bootstrap on the model (Lenth, 2021). We then determined the slope modifiers for species and storage from the estimated random effects (Appendix S2). Within this study, we defined the “suitable operational window” as the estimated time leaf chlorophyll takes to degrade to 95% of its original value. We selected a 5% threshold because it is practical and easy to interpret, while also being a good balance between overly restrictive and lenient values. As leaf chlorophyll decays with first‐order kinetics (Canjura et al., 1991), we calculated the time interval as:

$$t=\frac{\log0.95}{\log\left(1-\lambda \right)}$$

Where *t* is the suitable operational window, and *λ* is the change in chlorophyll content through time (i.e., the slope obtained from the chlorophyll degradation model). The smaller the slope value (λ) is, the faster leaf chlorophyll decays. We used the lower confidence interval of the calculated slopes for more conservative results.

The analyses involving the influence of traits on chlorophyll decay focused on mature leaves only. We extended the original model with no traits to include the three‐way interaction of each trait with time and storage, and removed the random slope for storage. We analyzed each trait individually, and then ran a model with all traits together minus the LMA, as it had a variance inflation factor >5 (James et al., 2013), plus its counterpart with no traits. We extracted the time effect from the single trait regression using *emtrends* (Lenth, 2021), and compared the net amount of variance explained by the use of three traits vs. none using *R* ^2^ (Nakagawa et al., 2017).

#### Chlorophyll variation study

We calculated the amount of variance explained in the chlorophyll content in mature leaves only, and in mature and expanding leaves. We used species, individual within species, leaf within individuals, and measurement position (i.e., within‐leaf level) on leaves as random effects, with variance proportions calculated using the intraclass correlation coefficient (Nakagawa et al., 2017).

The number of individuals and leaves per individual needed to obtain a result within 5% of the “true mean” of the species leaf chlorophyll content was determined following a three‐step process. First, we ran a mixed model for each species with individuals as the random effect to estimate the “true” leaf chlorophyll mean. We then calculated the leaf chlorophyll mean on a subsample composed of a combination of *n* individuals, leaves per individual, and measurements per leaf, with increasing sample sizes. This random draw was repeated 1000 times for each combination. Finally, we calculated the deviation between each subsample and the true mean. We defined as “suitable” a subsample with a deviation within 5% across 80% of subsamples in at least eight out of 10 of our study species. Based on the Bernoulli distribution, for proportions close to 80%, the estimates have a standard error of 1.26% when 1000 iterations were used to calculate these proportions.

We tested whether using one vs. three observations per leaf yielded different results in finding suitable subsamples using a generalized linear model with a binomial family using the package glmmTMB version 1.1.5 (Brooks et al., 2017). We used the proportion of suitable subsamples as the response variable and the number of individuals, number of leaves per individual, and number of observations per leaf as independent variables.

### Validation

We confirmed the accuracy of the spectral estimations by chemically extracting chlorophyll from one leaf each from nine individuals across four species, encompassing diverse chlorophyll contents and leaf characteristics. We also investigated leaf storage effects by examining four leaves from three of the species that were stored at room temperature for two days. The leaf collection and spectral analysis followed the same rules as the main fieldwork and took place in Perth, Western Australia (32°00′14″S, 115°53′49″E), while the lab analyses took place at Curtin University, Western Australia.

Leaves were initially scanned for spectrometric quantification, after which their chlorophyll was extracted using 100% methanol (Hurst Scientific, Forrestdale, Australia). We cut a 5‐mm‐diameter leaf disk as close as possible from the scanned area using a hole puncher. We immersed the disk in a few drops of methanol and ground it with a mortar and pestle until it was completely discolored. The resulting crushed leaf material was diluted with methanol to a final volume of 10 mL and filtered to remove solids. The filtered chlorophyll solution was stored in glass vials at 4°C until analysis within 1 h of extraction. The chlorophyll quantification was performed using a spectrophotometer (UV‐1280; Shimadzu, Kyoto, Japan), zeroing the absorbance readings at 750 nm and recording at 632.0 nm, 652.0 nm, and 665.2 nm (Porra, 2002; Ritchie, 2008). The total chlorophyll concentration was calculated using the trichroic equation according to Ritchie (2008).

We ran a linear regression analysis between the spectrometric chlorophyll index and chemically extracted chlorophyll content, including a term for interaction with the storage type (refrigeration vs. room temperature). We followed up with a 10‐fold three‐repeat cross‐validation using the R package caret version 6.0 (Kuhn, 2008) over the entire data set (leaves stored in refrigerator and at room temperature), and with the refrigerated leaves alone. We then extracted the validation outputs for the *R* ^2^ and root mean squared error (RMSE) values.

## RESULTS

**Figure 1:**
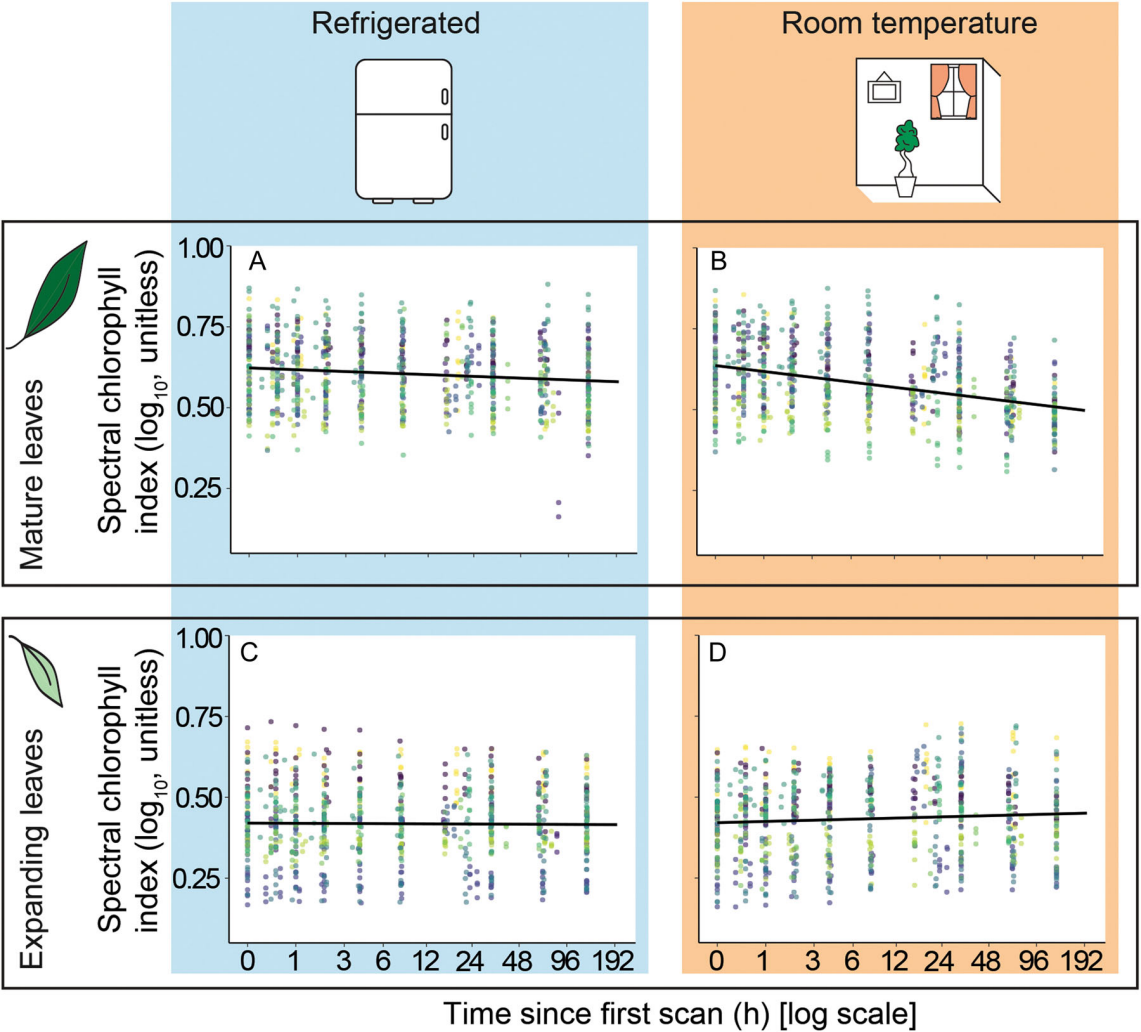
Chlorophyll decay in mature and expanding leaves stored in the refrigerator and at room temperature. (A, B) Chlorophyll decay in mature leaves under (A) refrigeration and (B) room temperature. (C, D) Chlorophyll decay in expanding leaves under (C) refrigeration and (D) room temperature. Each dot (●) represents an observation, and each color represents a species (for species trends, see Appendix S3).

The reflectance‐based estimates of chlorophyll content in mature leaves were found to take on average 4 h to decay to 95% of the original values. When refrigerated, the suitable operational window for mature leaves extended to 37.8 h (~1.5 days; *P* < 0.001). Although the operational window for the expanding leaves was also extended by refrigeration (*P* < 0.001), the chlorophyll showed virtually no decay within the study time (~5 days) regardless of the storage type (Figure 1, Appendix S3).

**Figure 2:**
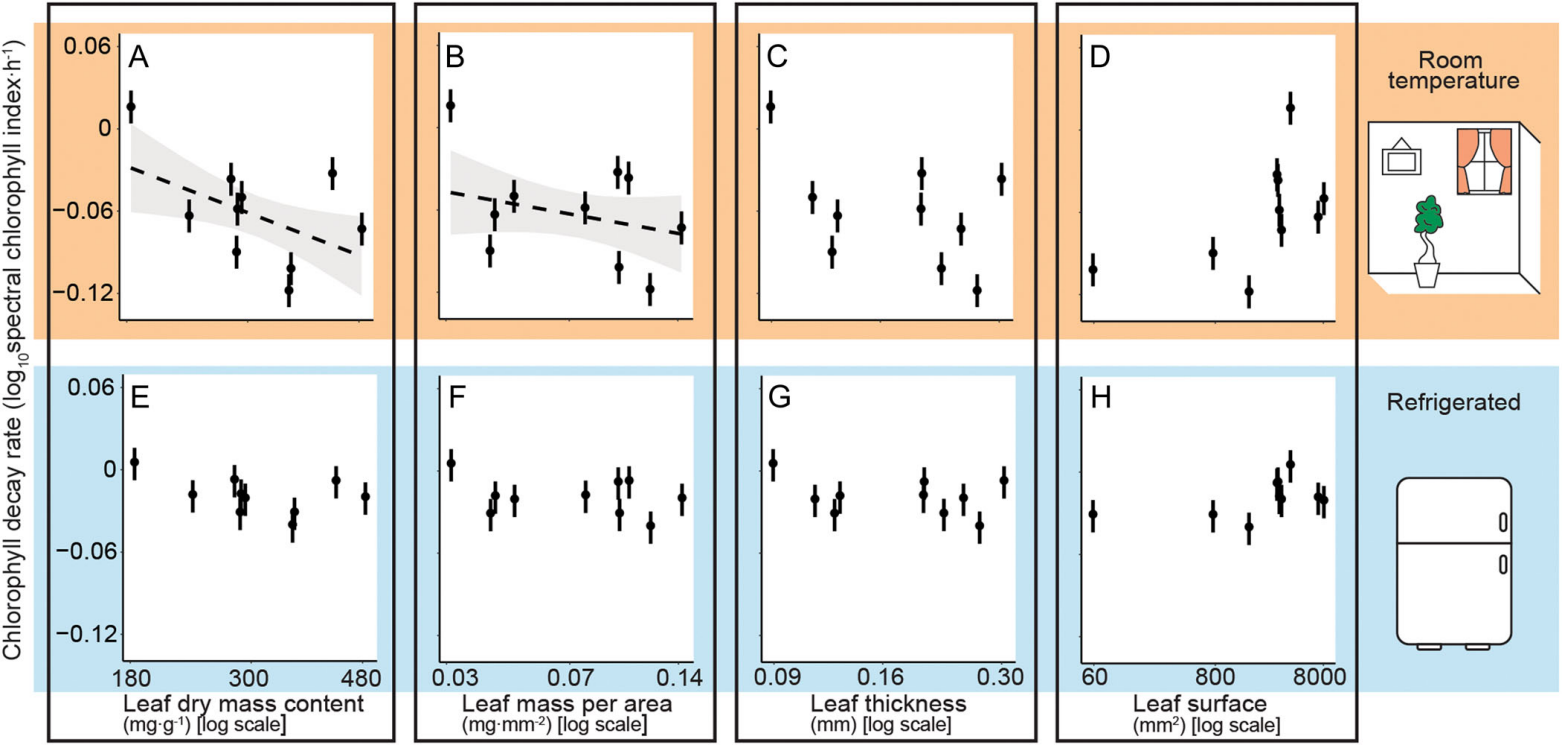
Relationships between leaf chlorophyll decay and leaf traits for each species. (A–D) Relationships between chlorophyll decay at room temperature and (A) leaf dry mass content, (B) leaf mass per area, (C) leaf thickness, and (D) leaf surface. (E–H) Relationships between chlorophyll decay under refrigeration and (E) leaf dry mass content, (F) leaf mass per area, (G) leaf thickness, and (H) leaf surface. Each observation is represented by dots (●) with a 95% confidence interval. More negative decay parameters represent faster chlorophyll decay. The shaded area is present only in the statistically significant relationships, A (*P* < 0.001) and B (*P* = 0.023), and expresses a 95% confidence interval.

We found no evidence that LDMC (*P* = 0.99), LMA (*P* = 0.95), leaf thickness (*P* = 0.94), or leaf surface area (*P* = 0.24) influence leaf chlorophyll decay when the leaves are refrigerated (Figure 2). At room temperature, leaves with a higher dry mass content (*P* < 0.001) or higher mass per area (*P* = 0.023) decay at a faster rate, while leaf thickness (*P* = 0.35) and leaf surface (*P* = 0.24) did not show significant relationships.

**Figure 3:**
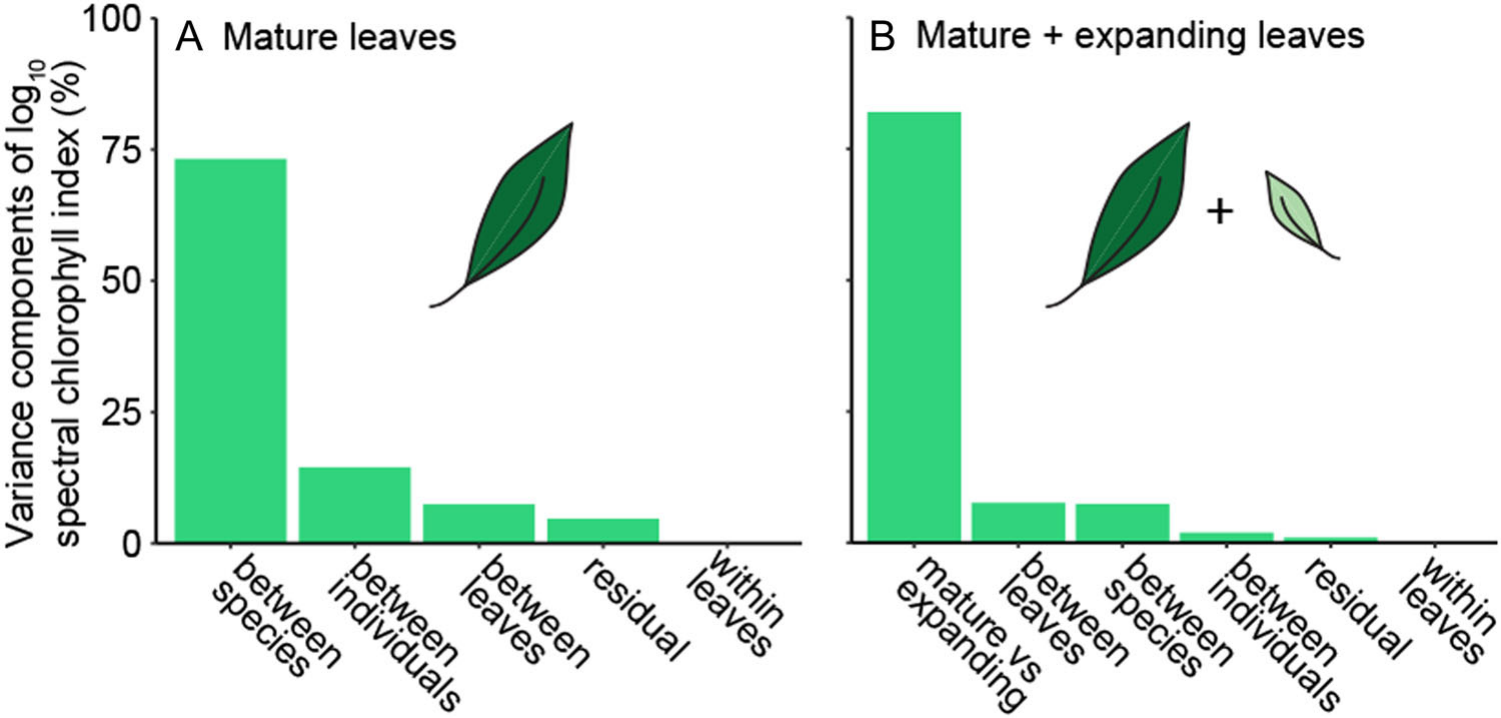
Amount of variance in chlorophyll content explained by the studied factors. (A) Variance in mature leaves. (B) Variance in mature and expanding leaves.

Most of the variation in the chlorophyll content of the mature leaves was described at the between‐species level (73.2%), with between‐individual (14.5%) and between‐leaf variation (7.5%) being much lower (Figure 3A). When considering expanding and mature leaves together, most of the variance in the chlorophyll estimate was explained by the growth stage (82.03%; Figure 3B).

### Most efficient allocation of sampling effort

**Figure 4:**
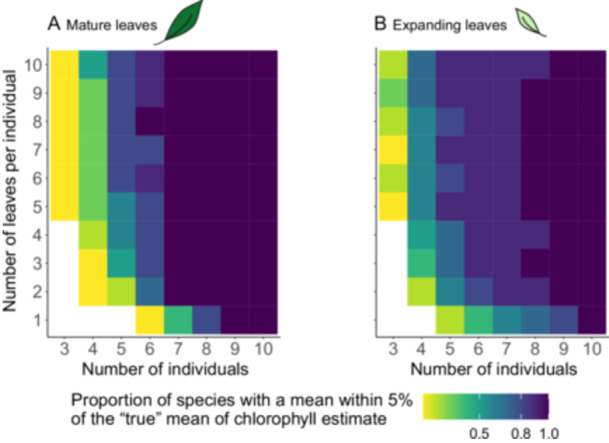
Combination of individuals and leaves per individual to guide the selection of an optimal estimate of the chlorophyll content measured via spectroscopy. (A) Combinations of mature leaves. (B) Combinations of expanding leaves. The colors represent the proportion of study species within 5% of the true mean with an 80% power for that particular combination, with darker hues representing higher proportions.

**Figure 5:**
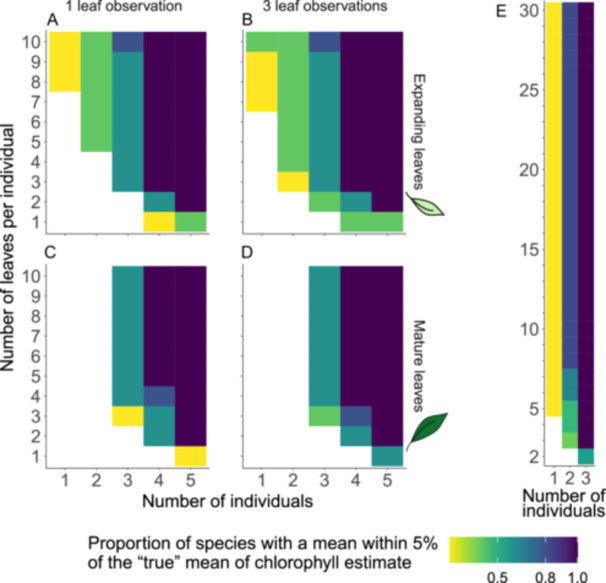
Combination of individuals and leaves per individual to select for a suitable chlorophyll content estimate measured via spectroscopy. For space reasons, only one (A, C) and three observations (B, D) per leaf are shown here. (A, B) Expanding leaves per individual. (C, D) Mature leaves per individual. (E) Subsample for one observation on up to 30 mature leaves per individual. The colors show the proportion of study species within 5% of the true mean with an 80% power for that particular combination, with darker hues representing higher proportions.

The most efficient way to estimate the chlorophyll content in a mature leaf within 5% of the true mean at 80% power is to measure a single leaf from each of eight individual plants (Figure 4A). For expanding leaves, sampling two leaves per individual for six individuals is advised for acceptable accuracy (Figure 4B). There seems to be no significant added information in measuring the same leaf more than once (Figure 5A–D; *P* = 0.26), nor when sampling up to 30 leaves from a single individual (Figure 5E).

### Validation

The chlorophyll estimates resulting from the spectrometric and chemical analyses were strongly positively correlated (adjusted *R* ^2^ = 0.83; *P* < 0.001). There was no evidence that storing leaves at room temperature for two days affected this correlation (*P* = 0.13). To convert spectral chlorophyll estimates to chemically extracted chlorophyll, one can use the formula:

$$\begin{array}{l} \text{spectral}\;\text{chlorophyll}\;\text{index}\;=2.16+1.11\\ \;\times \;\text{chemically}\;\text{extracted}\;\text{chlorophyll} \end{array}$$

**Figure 6:**
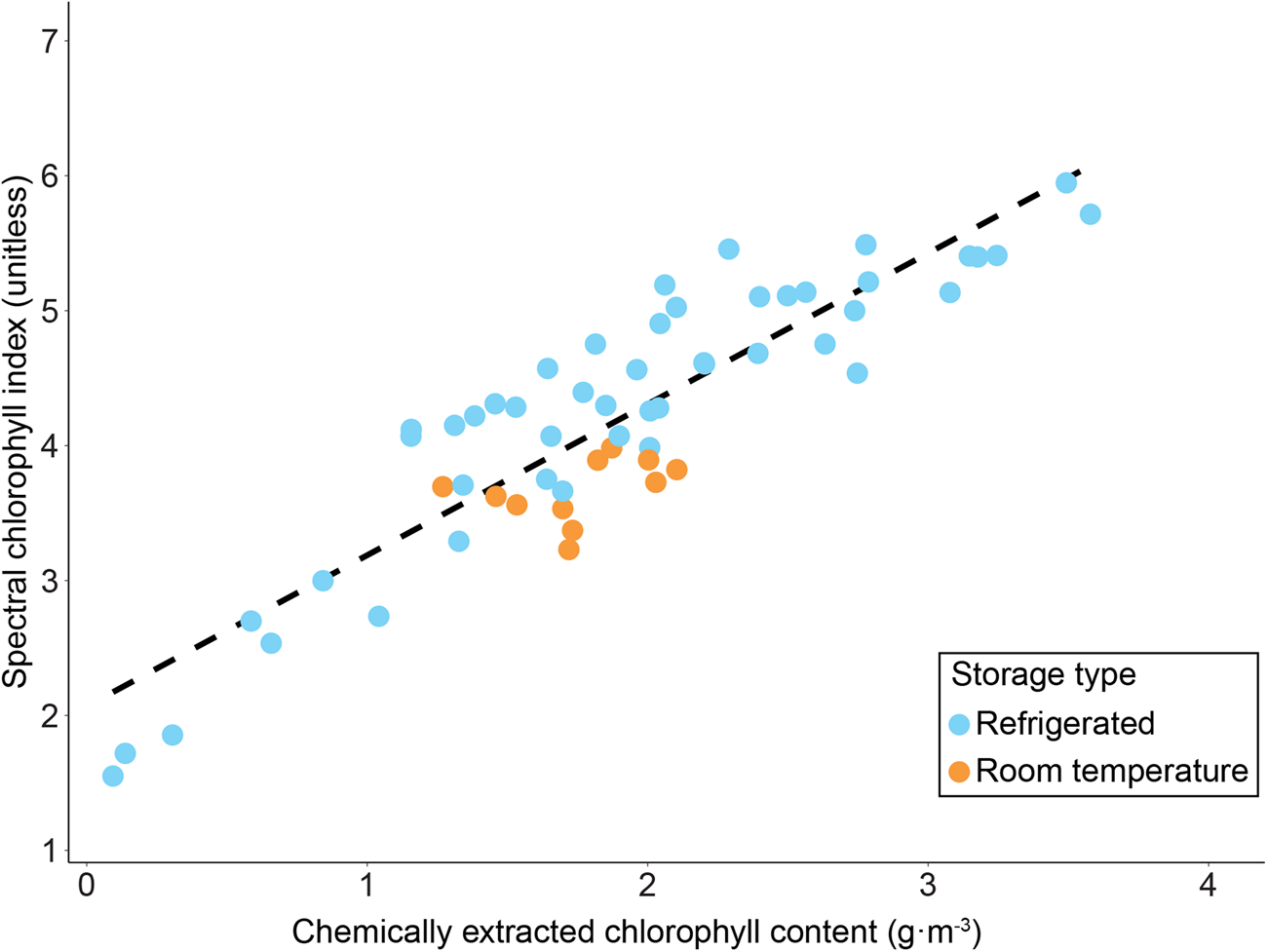
Correlation between spectral and chemical chlorophyll estimates. Adjusted *R* ^2^ = 0.834; *P* < 0.001.

For example, a spectral chlorophyll index of 4 would translate into approximately 1.65 g⋅m^−3^ of chemically extracted chlorophyll (Figure 6). The cross‐validation supported the robustness of the spectrometric readings. In fact, the cross‐validated model with only refrigerated leaves explained less than 2% more variance than the model with refrigerated and room‐stored leaves (*R* ^2^ = 0.866 vs. 0.852, respectively). In addition, the RMSE values for the models that included only refrigerated leaves and both storage types indicated little change in prediction error (0.424 and 0.393, respectively).

## DISCUSSION

Our study showed that ecologists aiming to quantify the chlorophyll content of mature leaves should invest their efforts towards sampling more species and individuals rather than measuring more leaves per individual or making multiple measurements per leaf (Figures 3 and 4). Most previous studies have made more measurements than the minimum requirement (Daughtry et al., 2000; Haboudane et al., 2002; León et al., 2007), meaning their findings are accurate, but most could have been achieved with less time and effort.

Refrigerating leaves effectively slows chlorophyll decay in mature leaves (Figure 1; *P* = 0.005), which aligns with the findings of Steet and Tong (1996). In our study, refrigeration increased the operational window by more than a day. Expanding leaves were still within the suitable operational window at the end of the five‐day experiment, regardless of storage type; however, keeping them refrigerated might help to delay the rotting process.

Leaf traits did not influence chlorophyll decay when the leaves were refrigerated (Figure 2); therefore, cooled leaves can be used to measure chlorophyll content within 1.5 days regardless of their characteristics. On the other hand, we observed that leaves stored at room temperature with a higher LDMC and LMA have a faster chlorophyll decay rate. It could be that such leaves might collapse faster after harvest because their structures are more demanding than flimsier leaves. In our study, both the refrigerated and room temperature leaves had access to the natural day/night cycle to reduce the confounding effect of light when analyzing the impact of storage type. Future studies on improving the sampling effort for leaf chlorophyll estimation should explore additional refrigeration temperatures (e.g., freezing), the effect of light on chlorophyll content kinetics, the role of leaf structures using internal analyses such as tomography, and effective ways to spectrally capture the chlorophyll index in leaves that are extremely hairy or spiny.

When collecting both expanding and mature leaves, we suggest measuring mature leaves first, but not delaying the measurement of expanding leaves for more than three days. While chlorophyll in mature leaves decays faster than in juvenile leaves (Figure 1), mature leaves can maintain their structural integrity for longer. Limited amounts of water loss due to storage in room conditions did not significantly affect the chlorophyll measurements (Figure 6), and we did not observe extreme dehydration in leaves during the recommended operational time period. Nonetheless, expanding and more delicate leaves, in particular, can crumple when stored at room temperature and tend to rot faster when damaged (Giancarlo M. Chiarenza, personal observation). This should also be taken into account when planning extensive fieldwork.

The chlorophyll index used in the guidelines is a reliable indicator of the chemically extracted chlorophyll content (Figure 6), which is in line with previous findings (Sims and Gamon, 2002; Mielke et al., 2012). The index ranged from 2 (low chlorophyll levels) to 7 (high chlorophyll levels), which closely mirrors the magnitude found in other studies (Sims and Gamon, 2002; Zhou et al., 2010; Mielke et al., 2012; Jin et al., 2012, 2016; Shrestha et al., 2023). Transmittance‐based chlorophyll meters, such as the Soil Plant Analysis Development (SPAD) chlorophyll meter (Konica Minolta, Tokyo, Japan) or the atLEAF chlorophyll meter (FT Green, Wilmington, Delaware, USA), are also strongly correlated with the chlorophyll content (Wood et al., 1993; Zhu et al., 2012); therefore, we believe our guidelines can be used across a wide range of techniques. Fieldwork is crucial in research, and an entire study can be compromised by inadequate sample collection. Leaves grow for limited periods and are easily damaged, so their collection must be as fast and efficient as possible. We hope this study will be used as a tool to help scientists decide on the most efficient sampling level, resulting in easier and cheaper data collection, thus allowing researchers to sample more species or more sites, or simply make data collection faster.

## AUTHOR CONTRIBUTIONS

G.M.C. and A.T.M. conceived the study idea; G.M.C., A.T.M., and E.S. designed the methodology; G.M.C. collected the data; G.M.C. and E.S. analyzed the data; G.M.C. and A.T.M. led the writing of the manuscript. All authors contributed critically to the drafts and approved the final manuscript for publication.

## Supporting information


**Appendix S1.** List of species collected and the study for which they were used.

**Appendix S2.** Details of chlorophyll degradation rates and operational windows for the study species.

**Appendix S3.** Chlorophyll degradation rate for the mature and expanding leaves of each study species, and for each storage type.

## Data Availability

Supplementary material, data sets, and code are available at https://tinyurl.com/ChlMethods.
