# Supplementary material for: Guidelines for quantifying leaf chlorophyll content via non‐destructive spectrometry
Source: Appl Plant Sci. 2024 Aug 3;12(6):e11610. doi: 10.1002/aps3.11610 (PMC11610418; doi:10.1002/aps3.11610)

DOI 10.1002/aps3.11610

Appendix S3. Chlorophyll degradation rate for the mature and expanding leaves of each study species, and for each storage type.

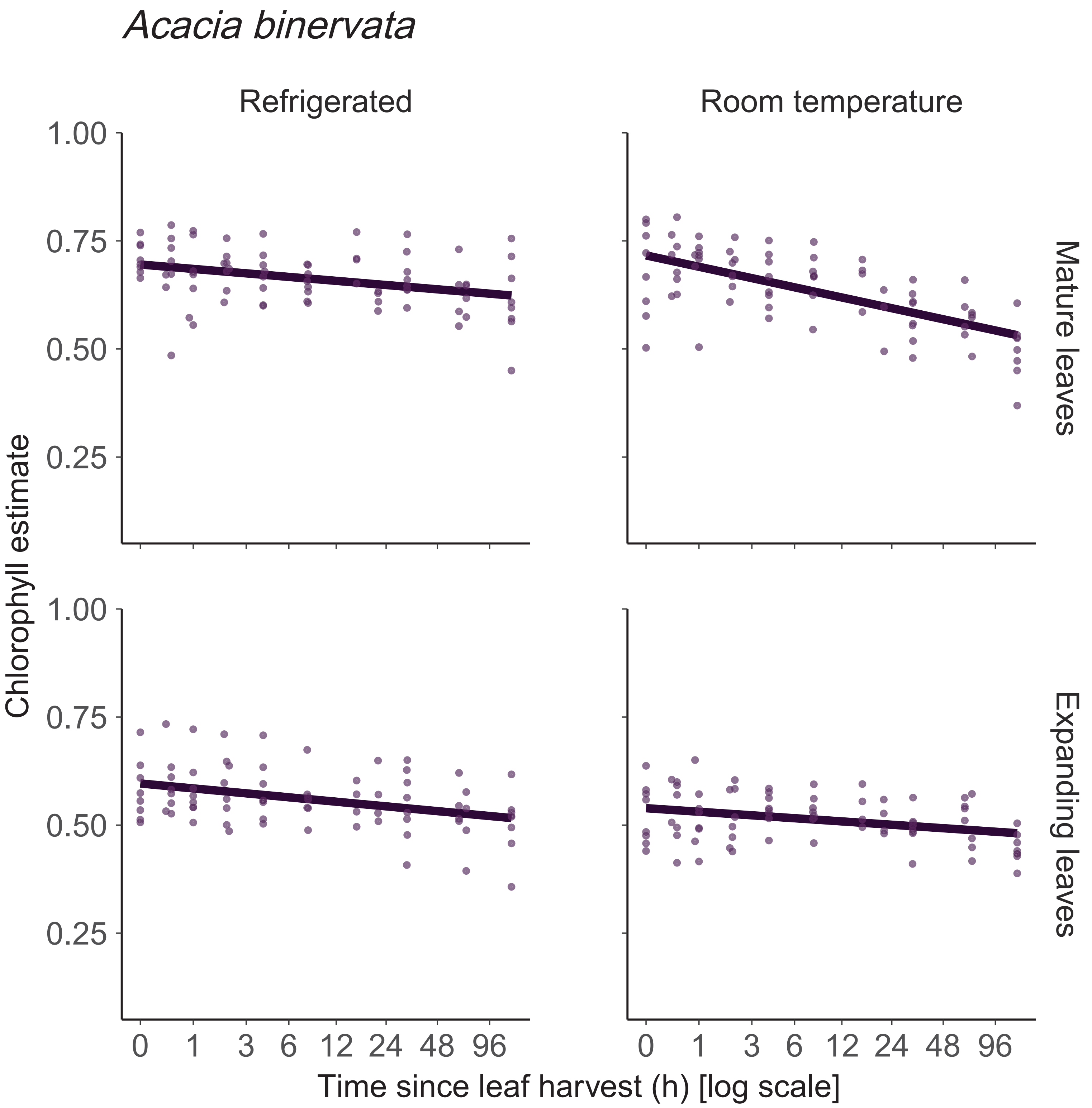

DOI 10.1002/aps3.11610

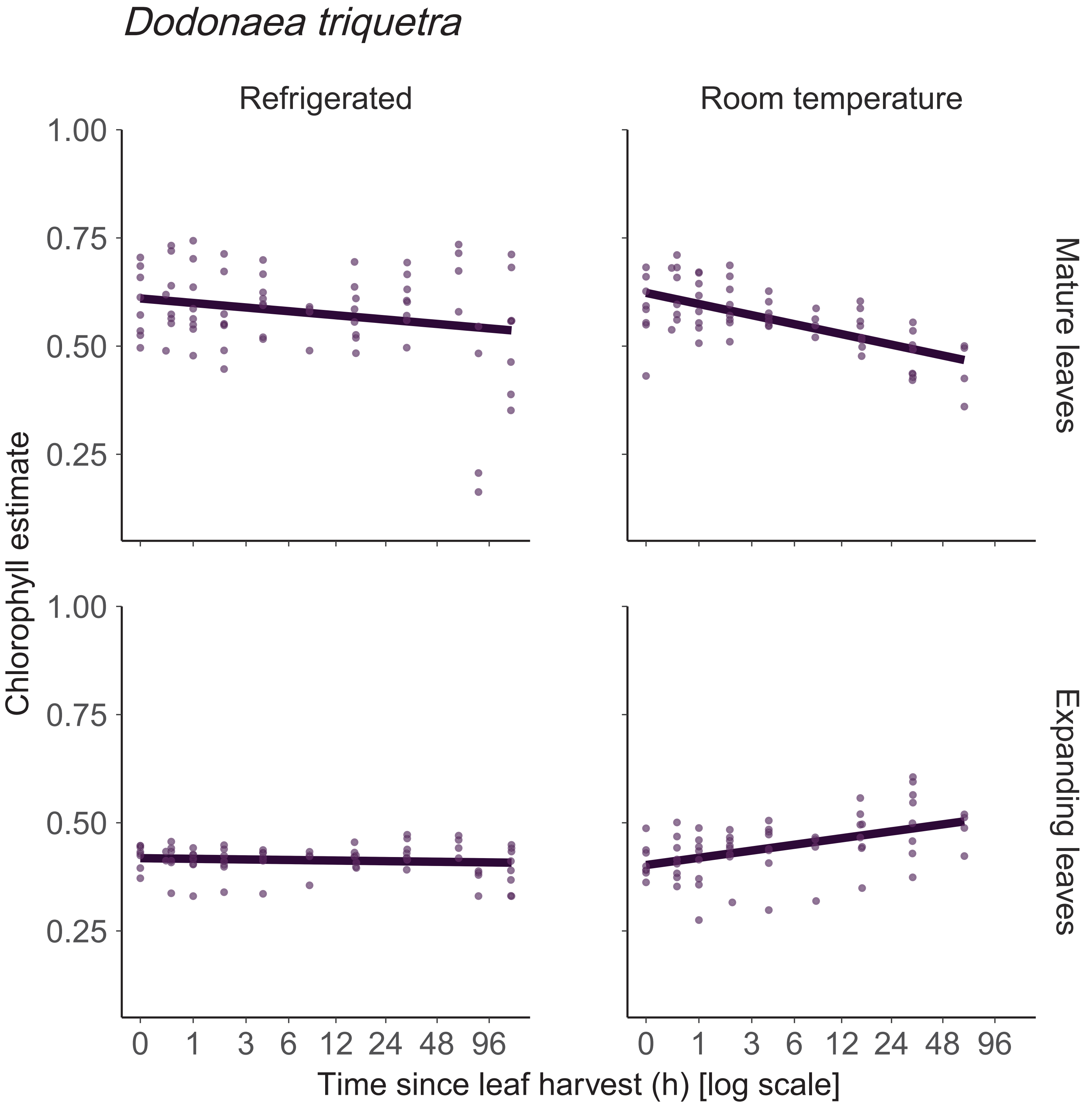

DOI 10.1002/aps3.11610

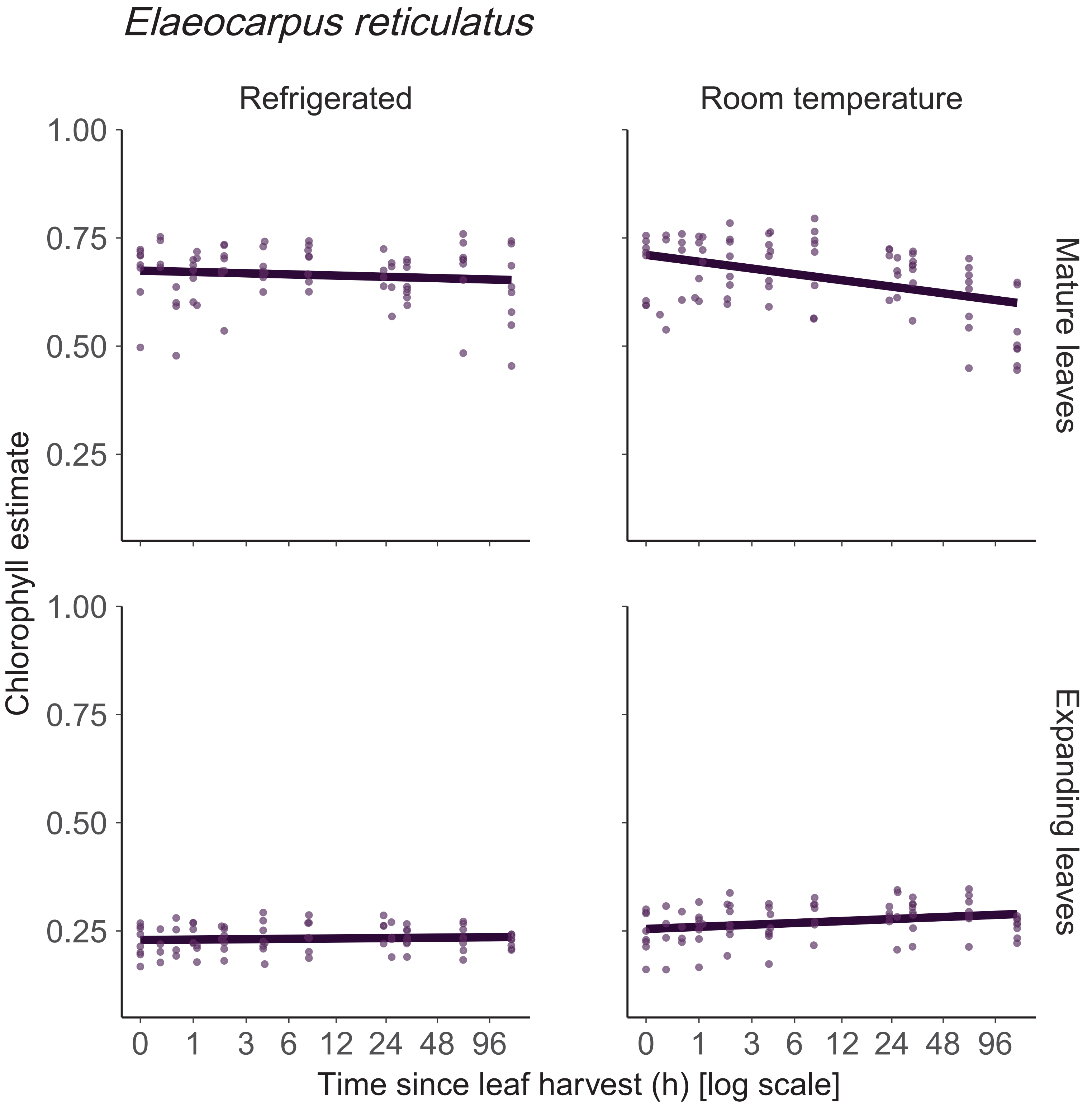

DOI 10.1002/aps3.11610

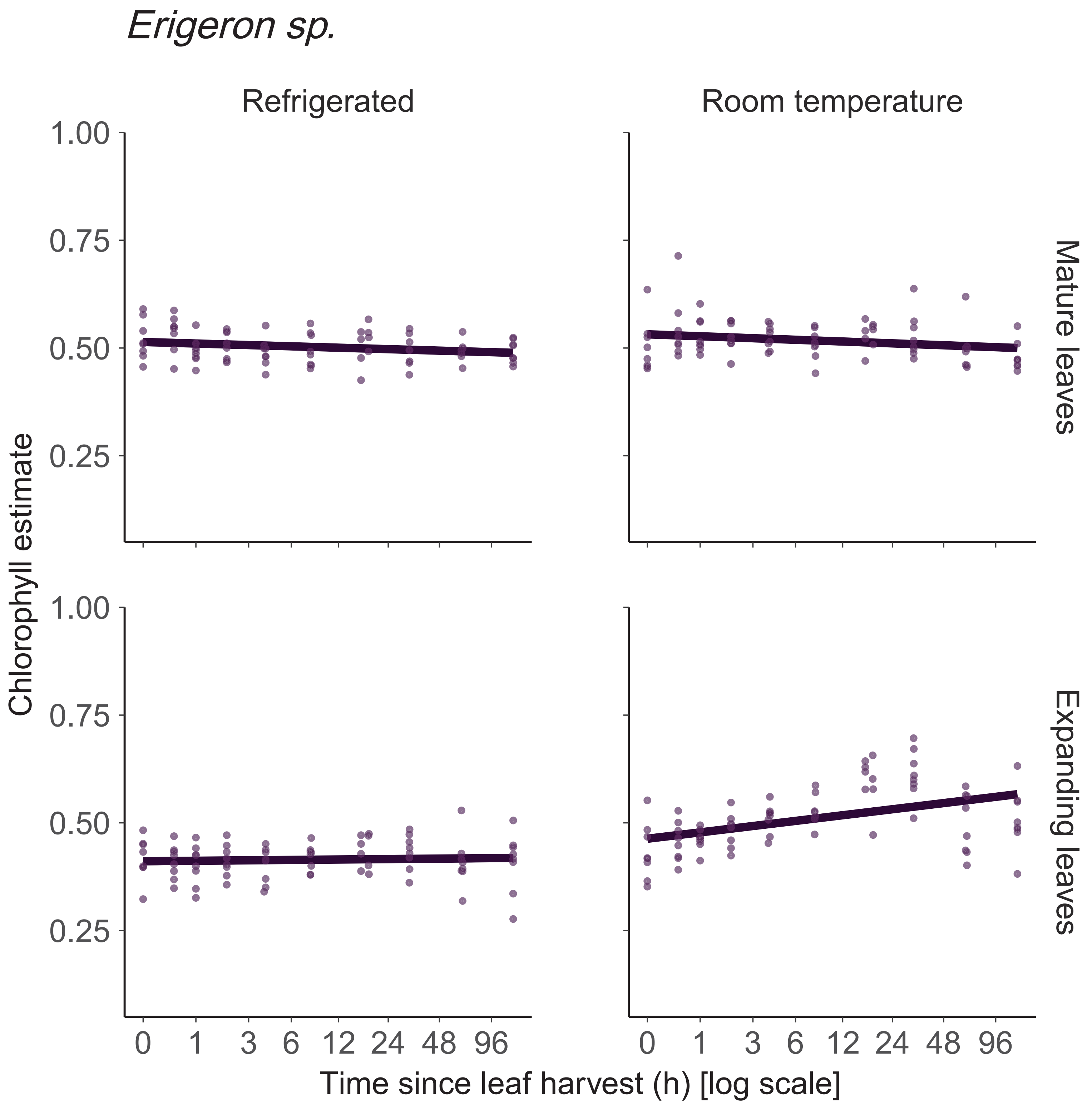

DOI 10.1002/aps3.11610

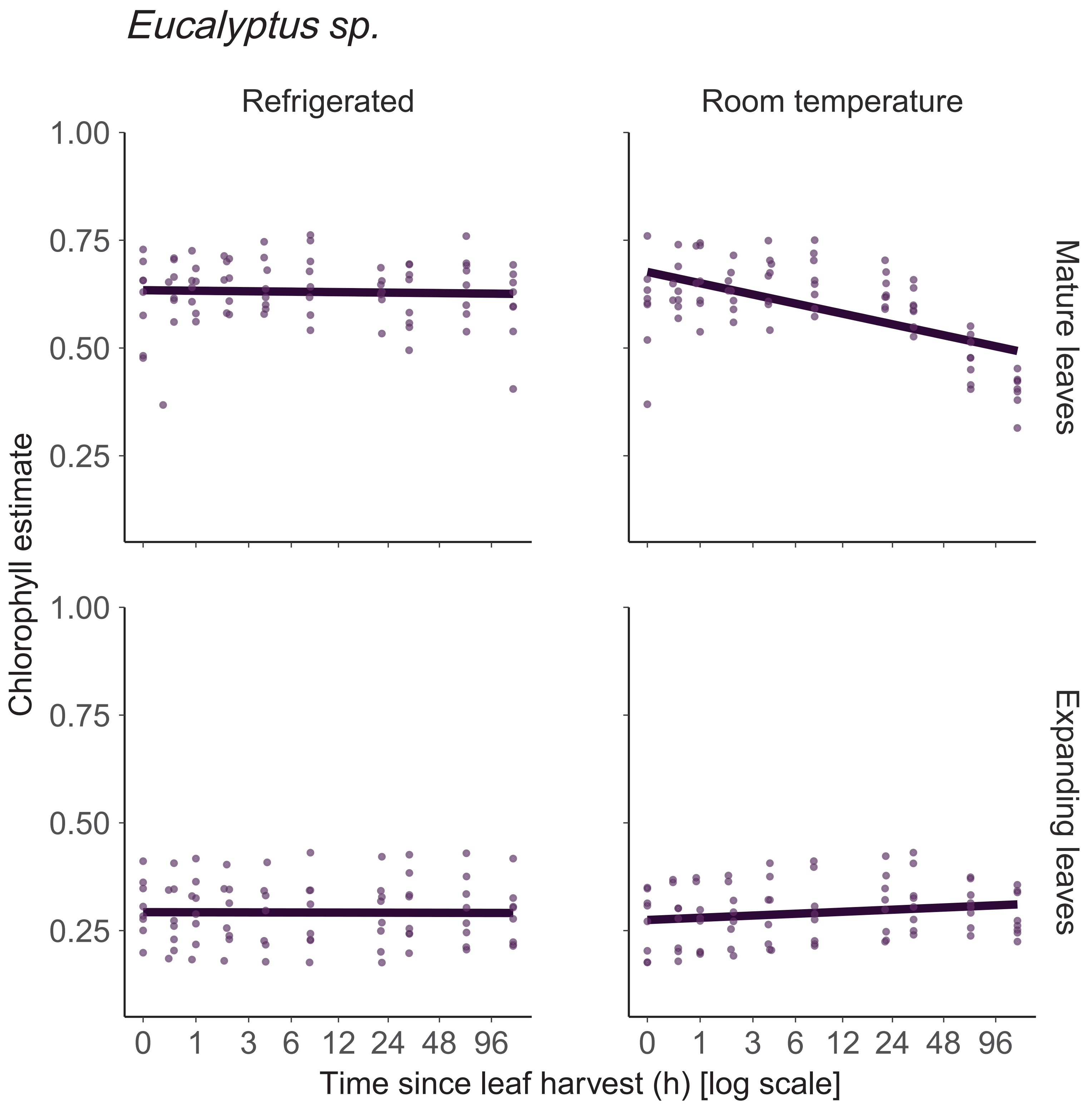

DOI 10.1002/aps3.11610

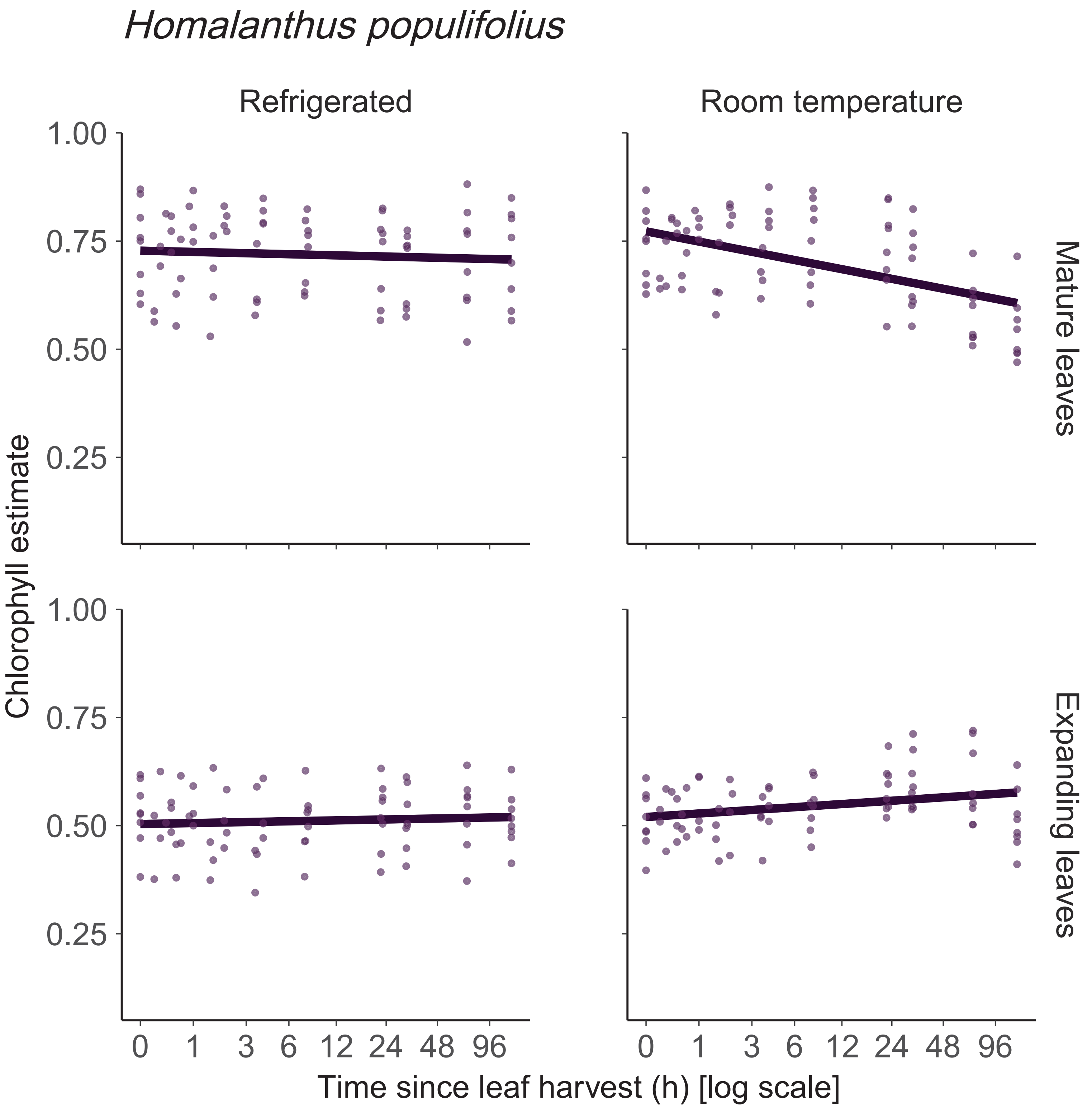

DOI 10.1002/aps3.11610

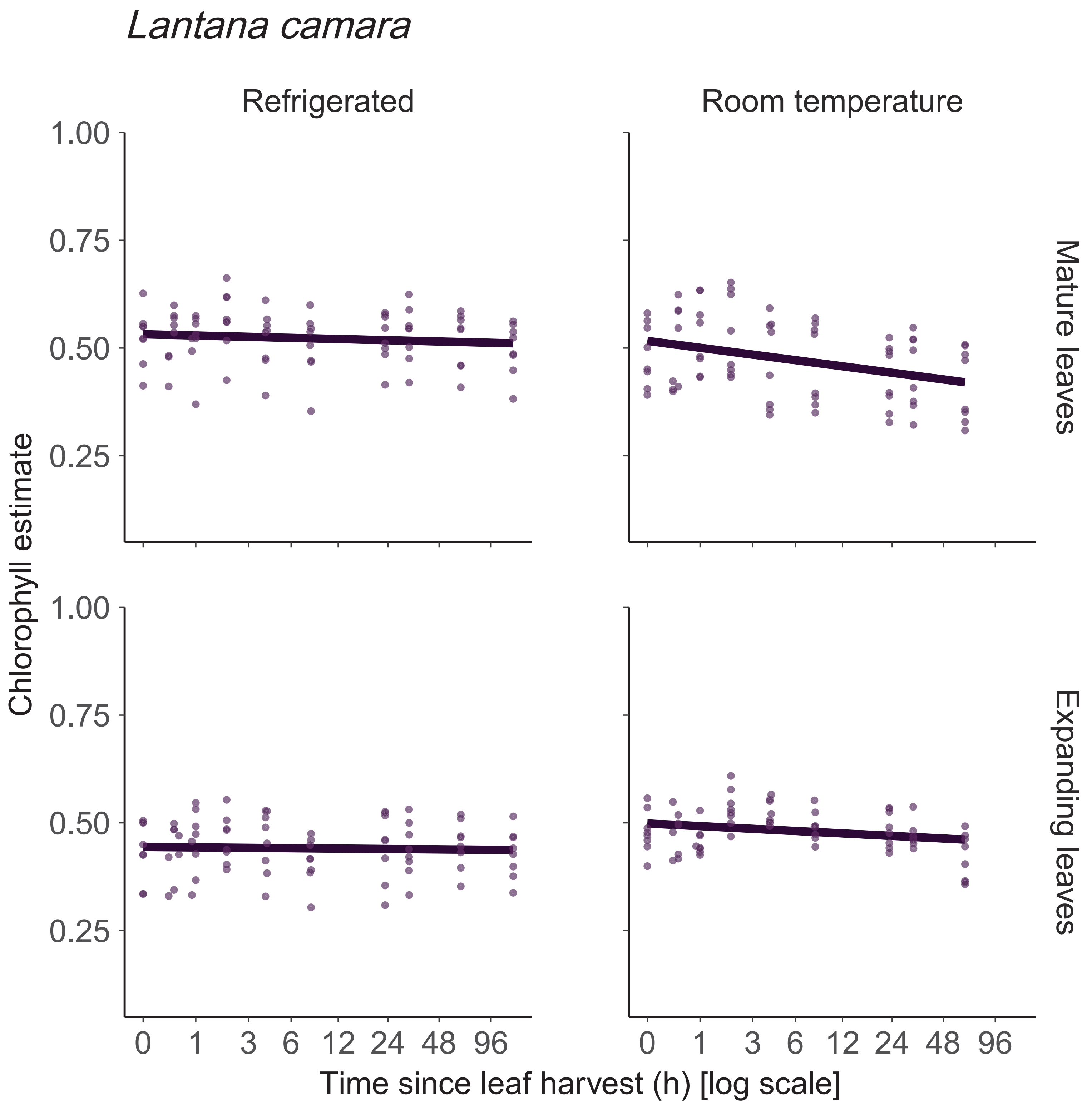

DOI 10.1002/aps3.11610

Leptospermum trinervium

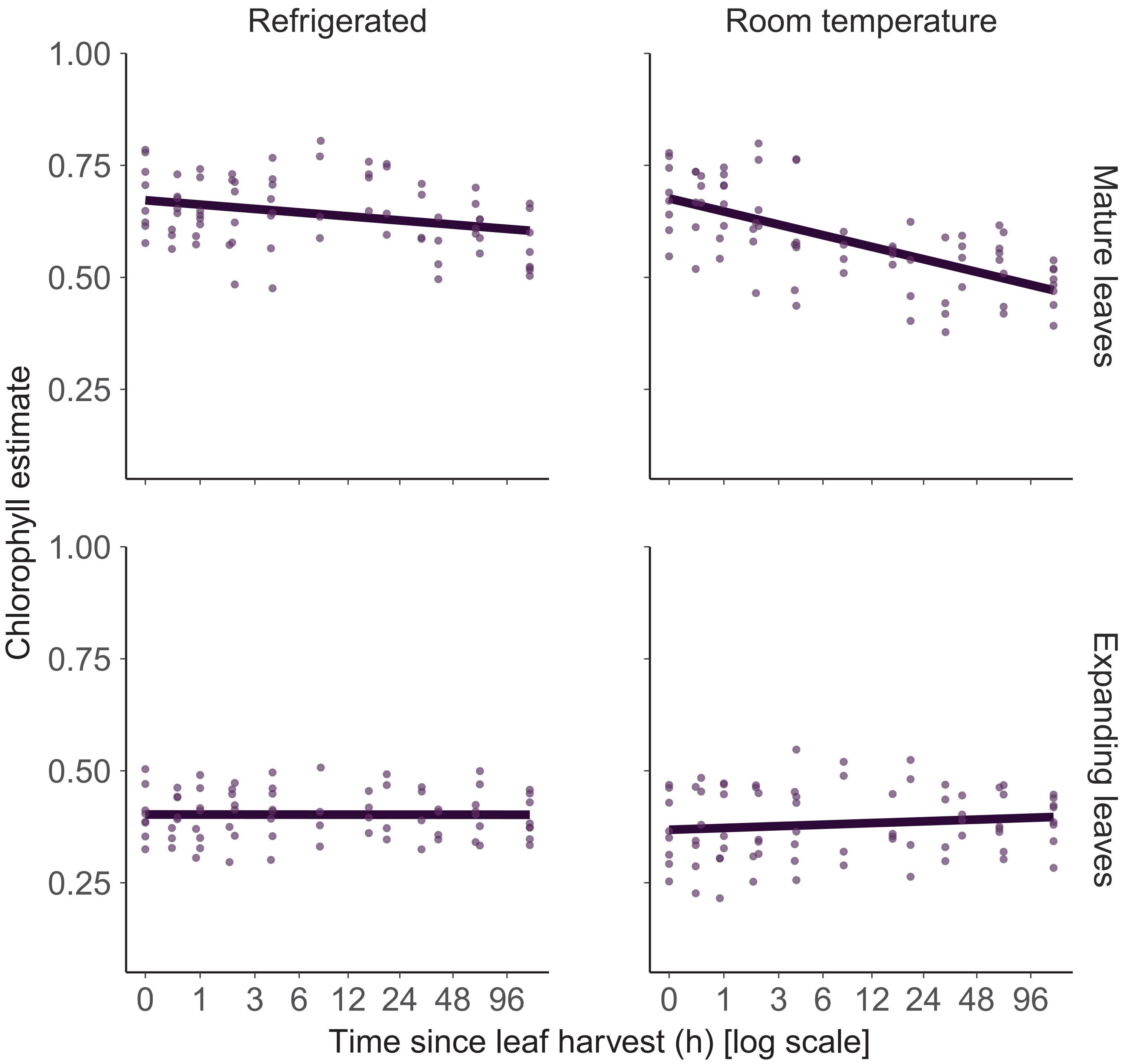

DOI 10.1002/aps3.11610

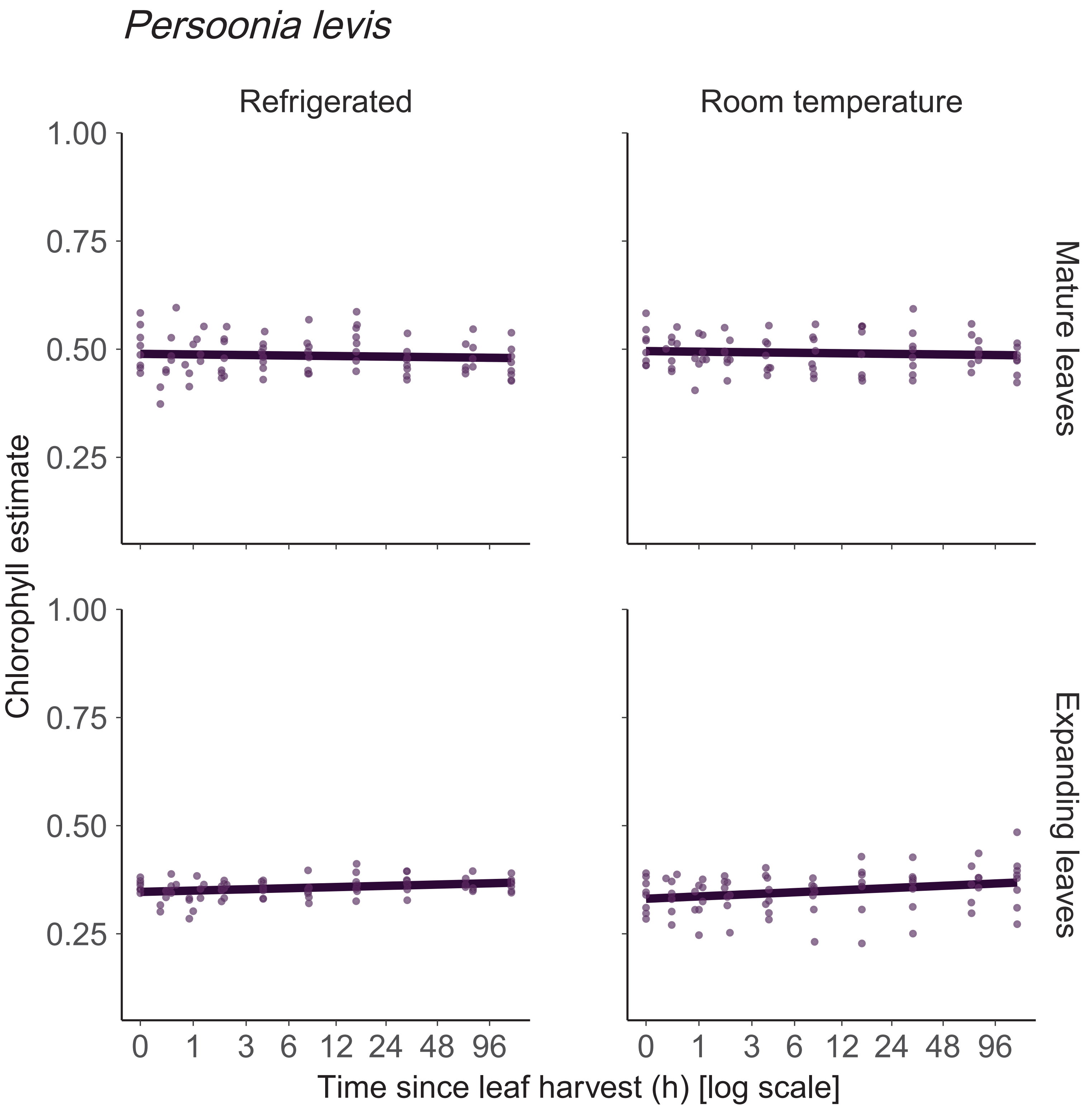

DOI 10.1002/aps3.11610

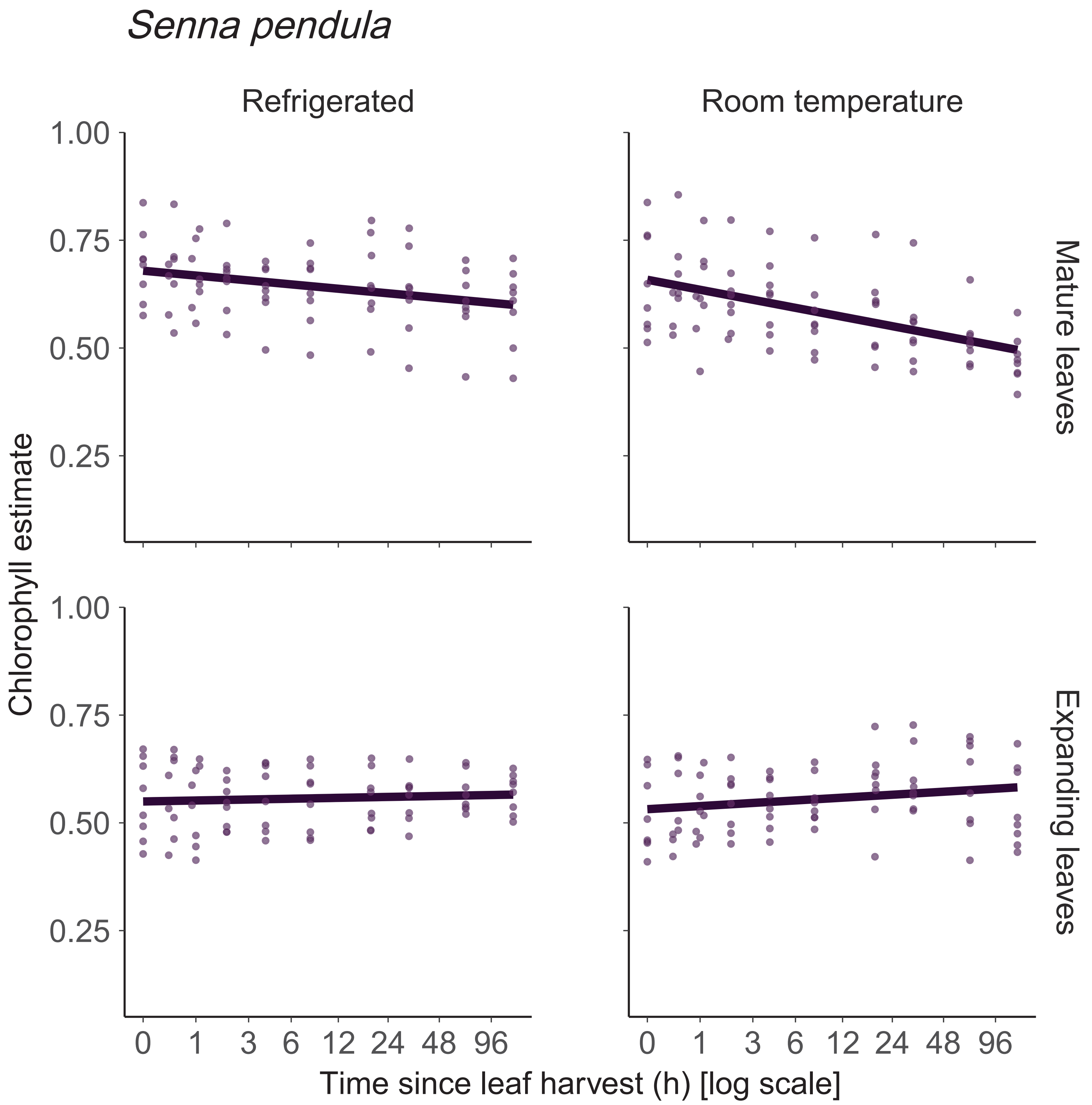

Supplement: Supplementary file 3 — Appendix S3. Chlorophyll degradation rate for the mature and expanding leaves of each study species, and for each storage type. [file APS3-12-e11610-s002.pdf]
